# Practice towards Hepatitis B Virus Infection Prevention and Its Associated Factors among Undergraduate Students at Hawassa University College of Medicine and Health Sciences, Hawassa, Sidama, Ethiopia, 2021: Cross-Sectional Study

**DOI:** 10.1155/2022/2673740

**Published:** 2022-08-12

**Authors:** Amdehiwot Aynalem, Bedilu Deribe, Mohammed Ayalew, Abyalew Mamuye, Eskinder Israel, Andualem Mebratu, Dawit Getachew Assefa

**Affiliations:** ^1^Hawassa University, College of Medicine and Health Sciences, School of Nursing, Hawassa, Sidama, Ethiopia; ^2^Wolaita Sodo University, College of Medicine and Health Sciences, School of Midwifery, Wolaita Sodo, SNNPR, Ethiopia; ^3^Dilla University, College of Medicine and Health Sciences, Department of Midwifery, Dilla, SNNPR, Ethiopia; ^4^Dilla University, College of Medicine and Health Sciences, Department of Nursing, Dilla, SNNPR, Ethiopia

## Abstract

**Background:**

Hepatitis B virus infection is a major global health burden accounting for 2.7% of all deaths globally. Being part of the health care system, the risk of exposure to hepatitis B viral infection among medical and health science students is found to be high. In Ethiopia, particularly in this study area, very little is known about the practice of students towards hepatitis B virus infection prevention and its associated factors.

**Objective:**

The aim of this study was to assess the practice towards hepatitis B virus infection prevention and its associated factors among undergraduate students at Hawassa University College of Medicine and Health Sciences, Hawassa, Ethiopia, 2021.

**Methods and Materials:**

An institution-based cross-sectional study was conducted from May 15 to June 15, 2021, among undergraduate students who had clinical exposure. The 404 sampled participants were recruited using a systematic random sampling technique. Data was collected using a structured self-administered questionnaire. Data was entered into EpiData version 4.6.0 and was exported to SPSS version 25 for analysis. Association between the dependent and independent variables was computed using the bivariate and multivariate logistic regression model. Odds ratio was calculated. Results were interpreted as significant if *P* value is <0.05 at 95% CI.

**Result:**

This study revealed that 277 (69.9%) of the students were in the age group of 20-24 years and 266 (67.2%) were males. Out of 396 participants, about half 199 (50.3%) 95% CI (0.452–553) had a good practice towards hepatitis B virus infection prevention. Only 43.4% of the study participants had been completely vaccinated against hepatitis B virus. Age (20-24 years) (AOR = 2.736), 95% CI (1.130-6.625), and good knowledge (AOR = 1.990), 95% CI (1.207-3.282) were factors significantly associated with the practice towards hepatitis B virus infection prevention. *Conclusion and Recommendation*. The current study showed that about half of the study participants had good practice towards hepatitis B virus infection prevention but more than half were not completely vaccinated against HBV. Age and knowledge were factors significantly associated. It is recommended to give training for students on hepatitis B virus infection prevention. It is also advisable to screen and vaccinate students before they start their clinical attachments.

## 1. Introduction

Hepatitis B virus infection is a major global health burden. It results in chronic infection and leaves people to die from cirrhosis and cancer of the liver. In 2019, the WHO estimated that 296 million people were living with chronic hepatitis B infection, with 1.5 million new infections each year and around 820, 000 deaths from cirrhosis and primary cancer of the liver (hepatocellular carcinoma) [1].

Health care workers are at high risk of acquiring HBV infection either through percutaneous injures (needle stick/sharp instrument) or mucosal exposures such as contact with nonintact skin, inappropriately sterilized instruments, and contact with infected blood/body fluids. The rise/incidence of HBV infection among health care professionals is four times greater than that in the general population [2].

Being part of the health care workforce, medical and health science students are at high risk of being infected with hepatitis B viral infection and spreading the infection because during clinical practice, their activities involve contact with blood or other body fluids during patient care, laboratories, or public-safety settings [3].

A study conducted among medical and health science students at Wollo University, northern Ethiopia reported that 4.2% of the study participants were positive for hepatitis B surface antigen (HBsAg). This study also reported that 50% of the students had poor practice towards hepatitis B prevention with a high prevalence of risky practices such as needle stick injury [4].

The study conducted in Woldia University health science students showed that 59.5% of the study respondents had poor practice towards HBV infection prevention and risky practices exposing to HBV infection were highly prevalent among the study participants [5].

Studies conducted at Gondar and Debre Birhan Universities, Ethiopia, also highlighted that there was a high risk of percutaneous and mucosal exposure among medical and health science students and also, there was poor compliance with the universal safety precautions such as usage of personal protective equipment [6, 7].

In this study area, very little is known about the practices of medical and health science students towards HBV infection prevention and its associated factors. This study was aimed at assessing the practice and its associated factors towards HBV infection prevention among undergraduate medical and health science students at Hawassa University College of Medicine and Health Sciences.

## 2. Methods and Materials

### 2.1. Study Design

An institution-based cross-sectional study was conducted.

### 2.2. Study Area

The study was conducted at Hawassa University College of Medicine and Health Sciences (HUCMHS). Hawassa University is a residential national university in Hawassa, Sidama Region, Ethiopia. It is approximately 278 kilometers (173 mi) south of Addis Ababa, Ethiopia. HUCMHS is one of the colleges found at Hawassa University. It has two faculties (Faculty of Medicine and Faculty of Health Sciences). There are 17 bachelors, 11 masters, 6 specialty, and 1 PhD programs in HUCMHS. There are around 3000 students who are currently studying at HUCMHS.

### 2.3. Source Population

All regular undergraduate students at HUCMHS who had clinical exposure during data collection period were the source population.

### 2.4. Study Population

All randomly selected regular undergraduate students at HUCMHS with clinical exposure were the study population.

### 2.5. Inclusion Criteria

The inclusion criteria were regular undergraduate students at HUCMHS who have clinical exposure, are available at the time of data collection, and volunteer to participate.

### 2.6. Exclusion Criteria

Students who were critically ill at the time of data collection were not included.

### 2.7. Sample Size Determination

The sample size was calculated by using single population proportion formula considering the following assumptions: confidence interval = 95%, critical value *Zα*/2 = 1.96, and a margin of error 5% (0.05). The estimated proportion was taken from a study conducted at Woldia University, Northeast Ethiopia, among health science students. From this study, 39.5% of the study participants had good practice towards HBV prevention [5], and 10% of the calculated sample size for possible nonresponse rate was added. The sample size calculated was 367. By adding 10% nonresponse rate, the final sample size was *n* = 404.

### 2.8. Sampling Technique and Procedure

First, students were stratified based on department and year of study. Second, the total sample size was distributed proportionally to each department based on their student proportion. Finally, using students' list, respondents were selected by systematic random sampling. *K* value was calculated (*K* = *N*/*n* = 1114/404 = 3). The first study unit was selected by lottery method. In case of absenteeism, the next number was included in the study.

### 2.9. Dependent Variable

The dependent variable is practice towards HBV infection prevention.

### 2.10. Independent Variables

#### 2.10.1. Sociodemographic Variables

- Age
- Sex
- Residence area
- Departments of study
- Ethnicity
- Year of study
- Marital status
- Religion

#### 2.10.2. Knowledge Towards HBV Infection Prevention

 

### 2.11. Operational Definitions

*Good practice*: if participants were able to score equal to or above the mean score of practice questions.

*Poor practice*: if participants scored less than the mean score of the practice questions.

### 2.12. Data Collection Instruments

A self-administered questionnaire was prepared by selecting, modifying, and adapting relevant and standard evaluation tools regarding the sociodemographic characteristics, knowledge, attitude, and practice towards hepatitis B virus infection prevention among students from different published literatures' written on a related topic of study [5–7]. The questionnaire was prepared in English.

### 2.13. Data Quality Control Measures

In order to keep the quality of the data, the questionnaire was pretested on 5% of the sample size one week before the actual data collection period. The data collectors were trained for two days on standardized data collection, and close supervision was done by the supervisors during data collection. The collected data were checked for completeness by supervisors on each day of data collection.

### 2.14. Data Processing and Analysis

The collected data was checked for completeness, coded, and entered into EpiData version 4.6.0 and was exported to SPSS version 25 for analysis. Bivariate and multivariate analyses were done to examine the relationship between the outcome variable (practice) and the predictor variables (sociodemographic factors, knowledge, and attitude). Variables that have a *P* value less than 0.25 upon bivariate analysis were entered into the multivariable logistic regression. Adjusted odds ratios (AOR) and their 95% confidence intervals (CIs) were used as indicators of the strength of association. Statistical significance was set at *P* value of less than 0.05.

## 3. Results

### 3.1. Sociodemographic Characteristics of Study Participants

A total of 396 students from nine departments participated in this study; from them, 277 (69.9%) of the study respondents were in the age group of 20-24 years and 266 (67.2%) were males. The mean age was 23.66 years SD (1.943) (Table 1).

### 3.2. Knowledge towards Hepatitis B Virus Infection Prevention among Study Participants

**Figure 1:**
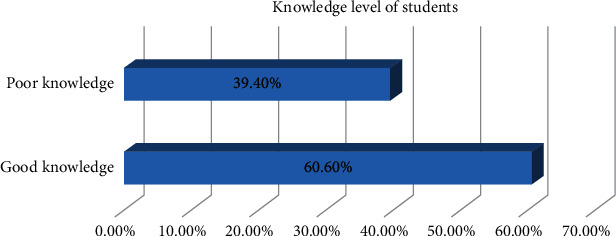
Knowledge level of students towards HBV prevention at HUCMHS, Hawassa, Ethiopia, 2021.

Out of 396 study participants, more than half (240 (60.6%), 95% CI (0.556–0.654)) had good knowledge towards hepatitis B virus prevention (Figure 1).

Of the students who responded, 370 (93.4%) knew that people infected with hepatitis B virus are at risk of infecting others and 366 (92.4%) knew that hepatitis B virus can be transmitted by unsterilized syringes, needles, and surgical instruments (Table 2).

### 3.3. Attitude towards Hepatitis B Virus Infection Prevention among Study Participants

Of 396 study participants, 222 (56.1%) (95% CI (0.51–0.61)) had a favorable attitude while 174 (43.9%) of the respondents had an unfavorable attitude towards hepatitis B virus infection prevention (Table 3).

### 3.4. Practice towards Hepatitis B Virus Infection Prevention

**Figure 2:**
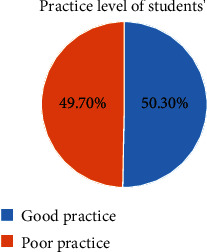
Practice level of students' towards HBV infection prevention at HUCMHS, Hawassa, Ethiopia, 2021.

About half 199 (50.3%) 95% CI (0.452–553) had good practice towards hepatitis B virus infection prevention (Figure 2).

Nearly three-fourth 296 (74.7%) of the respondents had been vaccinated against hepatitis B virus (Table 4).

Regarding the doses of HBV vaccine taken by the study participants, 172 (43.4%) of the study respondents reported that they had taken three doses of HBV vaccine but 99 (25%) and 25 (6.3%) of the participants responded that they had taken only two and one doses of HBV vaccine, respectively.

In this study, 54 (13.8%), 91 (23%), and 146 (36.9%) responded that reason for not being vaccinated, not being screened, and not using PPE is due to lack of resources, respectively (Table 5).

### 3.5. Factors Associated with the Practice towards Hepatitis B Virus Infection Prevention

In bivariate analysis, variables having a *P* value of less than 0.25 were considered as a candidate for multivariate analysis. Those variables were age, department, year of study, knowledge, and attitude towards hepatitis B virus infection prevention. In multivariate analysis, age of the respondents and knowledge were significantly associated with practice of the study participants. Those students at the age of 20-24 year (AOR = 2.736, *P* = 0.026, 95% CI (1.130-6.625)) were more likely to have a good practice towards HBV infection prevention than those aged from 25 to 29 years. Those who have good knowledge towards HBV infection prevention (AOR = 1.990, *P* = 0.007, 95% CI (1.207-3.282)) were also more likely to have good practice than those who have poor knowledge towards HBV infection prevention (Table 6).

## 4. Discussion

Exposure to hepatitis B virus infection remains a major occupational hazard to medical and health science students, especially in hepatitis B virus infection prevalent countries. In Ethiopia, very little is known concerning the practice towards hepatitis B virus infection prevention and its associated factors among medical and health science students. This study revealed the practice towards hepatitis B virus infection prevention and its associated factors among undergraduate medical and health science students at Hawassa University College of Medicine and Health Sciences.

Despite the wide variation of the professional background of the study participants, this study revealed that 50.3% 95% CI (0.452–553) of the study participants had good practice towards HBV infection prevention. The result from this research work is comparable with a study conducted among medical and health science college students in Saudi Arabia which indicated 47.2% of the study participants had good practice towards HBV infection prevention [8], and the study conducted at Wollo University medical and health science college which revealed 50% of the students had a good practice towards HBV infection prevention [4]. This may be due to similarity in study design and usage of similar data collection tools.

However, the finding of this study is higher than that of the study conducted at a medical college in Nepal among clinical year medical students which showed only 29 (14.2%) of the study respondents had good practice [9] and the study conducted at Woldia University College of Medicine and Health Sciences which showed 39% of the students had good practice [5]. Such discrepancy may be due to variations in perception of the study participants towards HBV prevention, clinical practice levels, educational curriculum differences, study area and sociocultural environment and resource availability, and/or allocation discrepancies in the study areas.

The finding from the current study is lower than that from the study conducted among medical students at Tanta University, Egypt, in which 68.1% of the students had good practice [10] and a study done among medical students of a tertiary care centre in Tamil Nadu, India, which reported 157 (76.59%) of the study participants had good practice towards hepatitis B virus infection prevention [11]. This may be due to variations in training practices, compliance status of students with the infection prevention and control guidelines, differences in educational background and hepatitis B virus epidemiology in the study areas, and inadequate facilities and equipment in the research area indicating the need to fill gaps by strengthening training on universal safety precautions and allocating resources.

Risky practices among the study respondents from the current study were highly prevalent, with 146 (36.9%) of them who had been exposed to a needle prick injury; and 138 (34.8%) had no intention to report the accident, and 125 (31.6%) does not always use personal protective equipment as necessary, and 61 (15.4%) does not dispose of wastes appropriately. This finding is higher than the study carried out at Gondar University in which 66 (26.8%) had sustained a needle prick injury and 132 (53.7%) had reported the incident (Abdela et al., 2016). The finding is also higher than the study result from Debre Birhan University in which 73.24% had not experienced a needle prick injury [7] and the study from Ghana among nursing students in which 57 (26.5%) had sustained a needle stick injury (Aniaku, Amedonu, and Fusheini, 2019), and regarding personal protective equipment utilization, the current finding is lower than the study from a tertiary care centre in Tamil Nadu, India, among medical students in which 94.6% use the necessary personal protective equipment during patient care [11] and a study from Debre Birhan University in which 82.54% reported that they always use personal protective equipment during patient care [7]. This may be due to lack of training, poor compliance with infection prevention and control guidelines, negligence of the study participants, and scarce personal protective items in the study area. And this finding suggests that there is a need to address the gap by strengthening health education on universal safety precautions for prevention of infections.

The current study showed that those students aged from 20 to 24 (AOR (2.736), 95% CI (1.130-6.625)) were more likely to have good practice towards hepatitis B virus infection prevention. This finding was in line with the study done at Woldia University, Ethiopia (AOR (1.396), 95% CI (0.185–10.560)) [5] and it is inconsistent with the study done at Debre Birhan University in which those aged 25-29 were more likely to have a good practice (AOR = 6.98; 95% CI (4.76-16.53)) [7]. This may be due to the reason that majority of graduating class students of the majority of the departments are found in this age group [6, 10, 12–14] because they are assumed to be competent in clinical practice like infection prevention, poor compliance with universal safety precautions of those aged from 25 to 29 years, and/or negligence/poor commitment of those aged from 25 to 29 years.

This study also revealed that those who have good knowledge (AOR = 1.990, 95% CI (1.207-3.282)) towards hepatitis B virus infection prevention were more likely to have a good practice towards HBV infection prevention. This finding is consistent with the study done among international students of University Putra Malaysia [13]. This may due to the fact that when the students' knowledge towards HBV prevention increases, the practice towards hepatitis B virus infection prevention will also increase.

## 5. Conclusion

Based on the finding from the current study, about half of the study participants had good practice towards hepatitis B virus infection prevention and more than half were not completely vaccinated against HBV. Age and knowledge towards HBV prevention were factors significantly associated with the practice towards hepatitis B virus infection prevention whereas lack of resource was the main reason for not being screened and vaccinated against hepatitis B virus.

## 6. Limitations of the Study

This study has the following limitations:

- The data was collected by using a self-administered questionnaire, and due to this, there could be a recall bias and social desirability bias of the respondents
- The study was limited to undergraduate students

## Figures and Tables

**Table 1:** Frequency distribution of sociodemographic characteristics of the study participants at HUCMHS, Hawassa, Ethiopia, 2021 (*n* = 396).

Variables	Category	Frequency	Percentage (%)
Gender	Male	266	67.2
	Female	130	32.8
Age	20-24	277	69.9
	25-29	119	30.1
Department	Medicine	220	55.6
	Public health officer	36	9.1
	Nursing	31	7.8
	Medical laboratory	28	7.1
	Midwifery	20	5.1
	Radiology	16	4.0
	Anesthesia	16	4.0
	Psychiatry	15	3.8
	Optometry	14	3.5
Residence	Urban	250	63.1
	Rural	146	36.9
Religion	Orthodox	188	47.5
	Protestant	121	30.6
	Muslim	68	17.2
	Others	19	4.8
Ethnicity	Oromia	158	39.9
	Amhara	98	24.7
	Sidama	55	13.9
	Tigray	40	10.1
	Others	45	11.4
Marital status	Single	377	95.2
	Married	19	4.8
Year of study	Third year	92	23.2
	Fourth year	140	35.4
	Fifth year	77	19.4
	Sixth year	87	22.0

**Table 2:** Frequency distribution of students' knowledge score towards hepatitis B virus infection prevention at HUCMHS, Hawassa, Ethiopia, 2021 (*n* = 396).

Variables	Responses	
	Yes *N* (%)	No *N* (%)
People who are infected with hepatitis B are at risk of infecting others	370 (93.4)	26 (6.6)
Can hepatitis B be caught through hand shaking?	178 (44.9)	218 (55.1)
Can hepatitis B be spread through contact with open wounds/cuts?	370 (93.4)	26 (6.6)
Hepatitis B can transmit from mother to fetus?	304 (76.8)	92 (23.2)
Hepatitis B can be transmitted through unprotected sexual intercourse	360 (90.9)	36 (9.1)
Can hepatitis B be transmitted by unsterilized syringes, needles, and surgical instruments?	366 (92.4)	30 (7.6)
Can hepatitis B be transmitted by contaminated blood and blood products?	375 (94.7)	21 (5.3)
Can hepatitis B virus cause liver cirrhosis?	349 (88.1)	47 (11.9)
Can hepatitis B virus cause liver cancer?	306 (77.3)	90 (22.7)
A person can be infected with hepatitis B and may not have any symptoms of the disease	305 (77.0)	91 (23)
Can HBV vaccine prevent hepatitis B?	351 (88.6)	45 (11.4)
Do you think hepatitis B virus has a laboratory test?	368 (92.9)	28 (7.1)
Is hepatitis B curable/treatable?	232 (58.6)	164 (41.4)
Do you think that HBV has postexposure prophylaxis?	320 (80.8)	76 (19.2)

**Table 3:** Frequency distribution of students' attitude score by Likert scale regarding HBV infection prevention at HUCMHS, Hawassa, Ethiopia, 2021 (*n* = 396).

Attitude test questions	SA *N* (%)	A *N* (%)	NA/ND *N* (%)	DA *N* (%)	SD *N* (%)
HBV is serious public health problem	116 (29.3)	191 (48.2)	40 (10.1)	42 (10.6)	7 (1.8)
I am not at risk of getting hepatitis B	46 (11.6)	51 (12.9)	27 (6.8)	172 (43.4)	100 (25.3)
I do not want to become screened for hepatitis B	16 (4)	21 (5.3)	25 (6.3)	176 (44.4)	158 (39.9)
I do not believe in the hepatitis B vaccine	16 (4)	22 (5.6)	19 (4.8)	157 (39.6)	182 (46)
Using personal protective equipment's during patient care and during carrying out procedures is a waste of time	42 (10.6)	40 (10.1)	25 (6.3)	124 (31.3)	165 (41.7)
I believe that instrument sterilization is important to prevent hepatitis B transmission	134 (33.8)	152 (38.4)	52 (13.1)	43 (10.9)	15 (3.8)
All patients should be tested for HBV before they receive health care	101 (25.5)	109 (27.5)	139 (35)	32 (8.1)	15 (3.8)
I do not feel comfortable to take care of people with HBV	35 (8.8)	90 (22.7)	68 (17.2)	140 (35.4)	63 (15.9)
I believe that following infection control guidelines will protect me from being infected with HBV at work	122 (30.8)	179 (45.2)	45 (11.4)	32 (8.1)	18 (4.5)
Vaccination towards hepatitis B is mandatory	111 (28)	174 (43.9)	65 (16.4)	29 (7.3)	17 (4.3)
Being a medical and health science student puts you at greatest risk of having HBV infection	107 (27)	204 (51.5)	41 (10.4)	22 (5.6)	22 (5.6)

Key: SA: Strongly agree; A: Agree; NA/ND: Neither Agree Nor Disagree; DA: Disagree; SA: Strongly Disagree.

**Table 4:** Frequency distribution of students' practice score regarding HBV infection prevention at Hawassa University College of Medicine and Health Sciences, Hawassa, Ethiopia, 2021 (*n* = 396).

Variables	Yes Number (%)	No Number (%)
Have you got screened for hepatitis B?	141 (35.6)	255 (64.4)
Have you got yourself vaccinated against hepatitis B?	296 (74.7)	100 (25.4)
I always use personal protective equipment's as indicated for procedures	271 (68.4)	125 (31.6)
I always keep my hand hygiene before and after all patient contact and before and after any procedure	308 (77.8)	88 (22.2)
Have you ever had a needle prick injury?	146 (36.9)	250 (63.1)
I do not recap needles after using them	229 (57.8)	167 (42.2)
I always discard sharp materials such as needle in the safety box	328 (82.8)	68 (17.2)
I always report for a needle stick injury	258 (65.2)	138 (34.8)
I discard wastes materials in hospitals on their appropriate container	335 (84.6)	61 (15.4)
Have you ever involved in an unsafe sex?	113 (28.5)	283 (71.5)

**Table 5:** Results of factors affecting the practice of respondents on hepatitis B virus infection prevention among medical and health science students at Hawassa University College of Medicine and Health Sciences, Hawassa, Ethiopia, 2021.

Factors	Frequency	Percentage
*Reasons for not being screened*		
Lack of resource	146	36.9%
Feeling of low risk status	67	16.9%
Lack of knowledge	42	10.6%
*Reasons for not being vaccinated*		
Lack of resource	54	13.8%
Busy schedule	25	6.3%
Feeling of low risk status	21	5.3%
*Reasons for not using PPE*		
Lack of resource	91	23.0%
Negligence	34	8.6%

**Table 6:** Bivariate and multivariate analysis of factors associated with practice towards HBV infection prevention among students' at HUCMHS, Hawassa, Sidama, Ethiopia, 2021.

Variables	Categories	Practice status		COR (95%)	AOR (95%)	*P* value
		Good practice	Poor practice			
Age	20-24	156	121	2.279 (1.463-3.549)	2.736 (1.130-6.625)	0.03^∗^
	25-29	43	76	1	1	
Department	Medicine	96	124	1	1	
	PHO	21	15	1.808 (0.885-3.693)	1.031 (0.405-2.623)	0.95
	Nursing	13	18	0.933 (0.436-1.998)	0.390 (0.144-1.060)	0.06
	Midwifery	13	7	2.399 (0.922-6.244)	1.245 (0.396-3.912)	0.70
	Psychiatry	10	5	2.583 (0.855-7.808)	1.458 (0.408-5.208)	0.56
	Anesthesia	11	5	2.842 (0.955-8.453)	1.497 (0.423-5.299)	0.53
	Radiology	11	5	2.842.9558.453	1.128 (0.328-3.871)	0.85
	Optometry	10	4	3.229 (0.983-10.612)	1.497 (0.380-5.896)	0.56
	Medical laboratory	14	14	1.292 (0.588-2.838)	0.530 (0.184-1.529)	0.24
Year of study	Third year	51	41	1	1	
	Fourth year	84	56	1.206 (0.708-2.053)	1.302 (0.701-2.418)	0.40
	Fifth year	30	47	0.513 (0.277-0.950)	0.615 (0.241-1.571)	0.31
	Sixth year	34	53	0.516 (0.284-0.935)	1.711 (0.508-5.762)	0.37
Knowledge	Good knowledge	92	64	1.787 (1.188-2.687)	1.990 (1.207-3.282)	0.01^∗^
	Poor knowledge	107	133	1	1	
Attitude	Favorable attitude	122	100	0.707 (0.475-1.053)	1.001 (0.610-1.642)	0.99
	Unfavorable attitude	79	95	1	1	

^∗^ *P* value < 0.05.

## Data Availability

All generated data are included in this article and its supporting document.

## Abbreviations

AOR: Adjusted odds ratio
CDC: Communicable disease control
COR: Crude odds ratio
CI: Confidence interval
ETB: Ethiopian birr
HBV: Hepatitis B virus
HCW: Health care worker
HU: Hawassa University
HUCMHS: Hawassa University College of Medicine and Health Sciences
IRB: Institutional Review Board
Km: Kilometers
PPE: Personal protective equipment
SPSS: Statistical Package for Social Science
WHO: World Health Organization.

## References

[1] World Health Organization (2020) (2020). *Hepatitis B factsheet.h Organization. Hepatitis B factsheet*.

[2] Coppola N., De Pascalis S., Onorato L., Calò F., Sagnelli C., Sagnelli E. (2016). Hepatitis B virus and hepatitis C virus infection in healthcare workers. *World Journal of Hepatology*.

[3] Al-Hazmi A. (2015). Knowledge, attitudes, and practice of medical students regarding occupational risks of hepatitis B virus in College of Medicine, Aljouf University. *Annals of Medical and Health Sciences Research*.

[4] Demsiss W., Seid A., Fiseha T. (2018). Hepatitis B and C: seroprevalence, knowledge, practice and associated factors among medicine and health science students in Northeast Ethiopia. *PLoS One*.

[5] Gebremeskel T., Beshah T., Tesfaye M., Beletew B., Mengesha A., Getie A. (2020). Assessment of knowledge and practice on hepatitis B infection prevention and associated factors among health science students in Woldia University, Northeast Ethiopia. *Advances in preventive medicine*.

[6] Abdela A., Woldu B., Haile K., Mathewos B., Deressa T. (2016). Assessment of knowledge, attitudes and practices toward prevention of hepatitis B virus infection among students of medicine and health sciences in Northwest Ethiopia. *BMC Research Notes*.

[7] Allene M. D., Delelegn G. G. (2020). Assessment of knowledge, practices and associated factors toward prevention of hepatitis B virus infection among students of medicine and health sciences in Debre Berhan University, NorthShewa, Ethiopia: a cross-sectional study. *International Journal of Surgery Open*.

[8] Saquib S., Ibrahim W., Othman A., Assiri M., Al-Shari H., Al-Qarni A. (2019). Exploring the knowledge, attitude and practice regarding hepatitis b infection among dental students in Saudi Arabia: a cross-sectional study. *Open access Macedonian journal of medical sciences*.

[9] Bhattarai S., Gyawali M., Sapkota S., Karki D., Lamsal S. (2020). Knowledge, attitude and practice of hepatitis B vaccination among clinical medical students at a medical college in Nepal. *Europasian Journal of Medical Sciences*.

[10] Atlam S. A., Elsabagh H. M., Shehab N. S. (2016). Knowledge, attitude and practice of Tanta University medical students towards hepatitis B and C. *International Journal of Research in Medical Sciences*.

[11] Vasantha Mallika M. C., Sivaanusuya S. (2020). Knowledge attitude and practice on prevention of hepatitis B infection among medical students of a tertiary care centre in Tamil Nadu, India. *International Journal of Research in Medical Sciences*.

[12] Adenlewo O. J., Adeosun P. O., Fatusi O. A. (2017). Medical and dental students’ attitude and practice of prevention strategies against hepatitis B virus infection in a Nigerian university. *The Pan African Medical Journal*.

[13] Ahmad A., Munn Sann L., Abdul Rahman H. (2016). Factors associated with knowledge, attitude and practice related to hepatitis B and C among international students of Universiti Putra Malaysia. *BMC Public Health*.

[14] Wibabara Y., Banura C., Kalyango J. (2019). Hepatitis B vaccination status and associated factors among undergraduate students of Makerere University College of Health Sciences. *PLoS One*.

